# Pooled bioequivalence study database from Turkey: characterization of adverse events and determination of split points based on Gini Index as a promising method

**DOI:** 10.1186/s40064-016-2527-4

**Published:** 2016-06-13

**Authors:** Çağrı Gurer, Ayça Çakmak Pehlivanli, Gonca Çakmak Demircigil

**Affiliations:** Turkish Medicines and Medical Devices Agency, Ankara, Turkey; Department of Statistics, Mimar Sinan FA University, Istanbul, Turkey; Department of Toxicology, Faculty of Pharmacy, Gazi University, Ankara, Turkey

**Keywords:** Bioequivalence, Adverse events, Gini Index

## Abstract

**Background:**

Manufacturing of and medication with generic drugs is increasing around the world. Bioequivalence (BE) studies are being performed routinely by Contract Research Organisations (CROs) in Turkey. However, an overall evaluation for the attended volunteers, examined Active Pharmaceutical Ingredients (APIs) and the observed adverse events have not been studied in the field.

**Objective:**

Our aim was to revisit and compile the BE studies carried out between the years 2000–2013 of a CRO (N-CRO) in Turkey.

**Methods:**

A dataset of 261 BE studies has been created for the observed adverse events with regards to the frequency, type, and drug subgroups. As an advanced evaluation, the Gini Index method has been used in the 63 available BE studies to obtain split points for two pharmacokinetic parameters, area under the plasma/serum concentration (AUC) and maximum plasma/serum concentration (Cmax), in order to investigate their likely effect on the adverse events.

**Results:**

Thousand six hundred and forty two adverse events were found in the 261 BE studies involving 7828 volunteers with the frequency of 6.29 per study and 0.21 per volunteer. The most frequently observed adverse events were; headache, somnolence, nausea, dizziness and vomiting, respectively. Hundred and nine different APIs were observed. ‘Genitourinary system and sex hormones’ subgroup drugs had the highest frequency of the adverse events. Adverse event frequencies above the identified split points for Cmax and AUC values were higher than the frequencies below them.

**Conclusion:**

The review of 13 years period of BE studies revealed that the demographic properties of the volunteers and the study designs were in compliance with national and international guidelines. The promising outcome could be showing the increase of the adverse event frequencies above the obtained split points as the reflection of the likely individual pharmacokinetic differences in the adverse event occurence.

**Electronic supplementary material:**

The online version of this article (doi:10.1186/s40064-016-2527-4) contains supplementary material, which is available to authorized users.

## Background

Generic drugs could be seen in the market as the copies of ‘originator’ drugs short after the different kinds of commercial protections have been expired (Kopp and Badiane 2014). Manufacturing and using of generics are constantly increasing worldwide because of cost efficiency, new formulation, and different administration route possibilities. Encouragement on development of generic drug manufacturing could be assumed as innovation and investments (Kopp and Badiane 2014; OECD 2015). Generics should provide pharmaceutical quality similar to the originator drug framed by regulatory guidelines (USFDA 2003; EMA 2010). National and international health authorities stipulate that BE studies are mandatory for the registration of the generics (European Parliament 2001; Ucar 2009).

Bioavailability (BA) and BE terms started to be used in late 60s and in early 70s. In 1984, US Congress established the Drug Price Competition and Patent Term Restoration Act (Public Law 1984; Midha and McKey 2009). Under this Act, Food and Drug Administration (FDA) had a crucial role on statutory generic drug approval process. Since 2003 the BE studies in USA have been conducted according to the fundamental guide named “Bioavailability and BE Studies for Orally Administered Drug Products-General Considerations” which has been published by FDA (USFDA 2003; Midha and McKey 2009). When it comes to European Union, European Medicine Agency has revised the regulatory guideline for BE studies in the name of ‘Guideline on The Investigation of Bioequivalence’ in 2010 which was first published as ‘Investigation of Bioavailability and Bioequivalence’ in 1991 (Commission of the EC 1991; EMA 2010). In Turkey, the directive of “Investigation of Bioavailability and Bioequivalence of Pharmaceutical Preparations” has been in use in concordance with the European Union since 1994 (Investigation 1994).

Aronson and Ferner (2005) defined the adverse events as ‘any abnormal sign, symptom, laboratory test, syndromic combination of such abnormalities, untoward or unplanned occurrence (e.g. an accident or unplanned pregnancy), or any unexpected deterioration in a concurrent illness’. When the adverse event causes death, life threatens, requires inpatient hospitalization or prolongs the existing hospitalization, results in persistent or significant disability/incapacity, or is a congenital anomaly/birth defect, then it is called serious adverse event (ICH GCP 1996). If any untoward medical occurrence that at any dose: results in death, is life-threatening, requires inpatient hospitalization or prolongation of existing hospitalization, results in persistent or significant disability/incapacity, or is a congenital anomaly/birth defect, is named as serious adverse event. (ICH GCP 1996). According to the all regulatory guidelines mentioned, it is mandatory to collect and verificate the reports of adverse events/reactions in BE studies and submit them periodically to the health authorities (USFDA 2009; European Commission 2011).

Although BE studies are being frequently carried out under the well established guidelines by CROs all over the world, the authors could not find a study with regards to the overall detailed evaluation of the adverse events observed in the series of BE studies. All the available scientific publications on the drug adverse reactions were mostly focused on single BE studies, the Phase I clinical trials and marketed drug products (Lazarou et al. 1998; Sibille et al. 1998; Baker et al. 2004). Hence, the review of the adverse events of pooled BE studies could provide valuable scientific data.

Herewith, our aim was to revisit the registered BE studies of a CRO (N-CRO) in Turkey between the years 2000–2013 for an overall statistical evaluation regarding the type, frequency, drug subgroups, cut off (split points using Gini Index) values and probable causes of adverse events.

## Methods

### Procedure at the investigator’s site

Each BE data used in this study was carried out with the collaboration of N-CRO and Clinical Research Centers between 2000 and 2013 and conducted according to Clinical Study Protocol prepared by Clinical Research Department of N-CRO. Accordingly, N-CRO strictly complied and followed the national and international guidelines in the selection of the volunteers in terms of age and body mass index and in the BE study design. The guidelines mandate; subjects should be 18–55 years of age and preferably have a Body Mass Index (BMI) between 18.5 and 30 kg/m^2^ (Investigation 1994; EMA 2010). In the N-CRO, design of the BE studies were carried out as classical two periods, replicated three periods, or four periods design according to FDA (USFDA 2003). Recommendations of FDA have been followed for adequate washout period (more than five half-lives of the drug), amount of water for drug administration (240 ml), and water/food restriction/allowance of subjects (e.g. water is restricted for 1 h before and after drug administration, standard meals should be given no less than 4 h after drug administration). Each subject attended to the study signed Informed Consent Form. Information of the adverse events observed during the study was recorded to the “Adverse Event Form” or “Serious Adverse Event Form” by the investigator. The MedDRA dictionary based adverse event codification was not obligatory while the BE studies were carried out and we had used the classification methodology of investigators of Clinical Trial Centers. We acquired a written permission from N-CRO dated 26.06.2013 and numbered 165 which allows us to use database of the BE studies between the years 2000–2013. The authors had full access to the data of BE studies including the demographic data of the volunteers according to the informed consent forms those signed by each volunteer.

### Dataset creation of the pooled BE studies

Dataset used in this paper included 261 BE studies with 7828 healthy subjects (i.e. volunteers). Each study had different number of subjects such as 24, 36, 48 and each subject had some features (i.e., variable) such as demographic information, existence/absence information of adverse event, types of adverse events, period (the process that starts with administration of test or reference drug and ends up with taking last biological sample) that adverse event was observed. The adverse event names were taken from the Adverse Event Forms as the investigator registered. In addition to these features, there were also two pharmacokinetic parameters named AUC and Maximum Plasma/Serum Concentration Time Curve From Zero up to the Last Quantifiable Concentration (Cmax) accessible for 63 studies done after 2005. All these features were extracted from the N-CRO Bioanalysis Laboratory Database and combined into a single data matrix. Drugs that were used in these BE studies and their subgroups that correlated to ATC were also added to the dataset. These codes were assigned based on World Health Organization Collaborating Center for Drug Statistics Methodology (WHO 2015). Preprocessing period which is essential before all the analysis was conducted in order to clean the raw data. During this essential phase, different units that belong to the same feature were converted to the same unit. Since there were no missing and duplicated values in the data set, no deletion has been carried out. Beside these, although there were extreme values detected by SPSS in the raw data, no extreme values observed after logarithmic transformation.

In the scope of this study, all analysis can be considered in twofold: statistical analysis and Gini Index approach. In the dataset, only 63 BE studies with 1992 subjects out of 261 BE studies had Cmax and AUC values with their reference and test values available. While all the demographic informations and descriptive statistical analysis has been carried out for the 261 BE studies, Gini Index calculations and other statistical analysis have been carried out with those 63 BE studies.

### Statistical analysis

Besides the descriptive statistics (mean, variance, min–max values), hypothesis tests for the assumptions including normality and homogeneity of variance were performed using SPSS version 21. Kolmogorov–Smirnov test was utilized to check whether the variables Cmax (test and reference) and AUC (test and reference) follow a normal distribution at 5 % level of significance. As a result of this test, neither Cmax nor AUC distributed normally (significance level = 0.05). Since the distribution of the AUC and Cmax tend to be skewed and their variances increase with the means, log transformation was used. Therefore the variances became independent of the mean and the distributions tend to be more symmetric (Rani and Pargal 2004). All analyses were done by using logarithmically transformed Cmax and AUC values. According to experimental design, each subject takes both test and reference drugs in different periods for all studies. In order to compare these two related groups (test or reference) for each variable (Cmax or AUC), dependent t test called paired sample t-test were conducted at 5 % level of significance.

### Gini Index

It should be noted that all calculations were done for both Cmax and also AUC values, but in order to save spaces only explanations related to Cmax will be given. It is aimed to obtain a split point which shows possible relationships between Cmax values and adverse event exist/not-exist knowledge. In the concept of this study, it is important to obtain a split point denoted *v* that divides dataset into homogenous subsets according to knowledge of “there is adverse effect” and “there is no adverse effect”. Once *v* is found, it is assumed that dataset is split into two homogenous, i.e. pure classes based on adverse event (exists/not exists) such that values lower than *v* (Cmax ≤ *v*) or values higher than *v* (Cmax > *v*). In order to find $v$, Gini Index method was preferred, and used with Cmax values of 1992 subjects from 63 BE studies by combining their test and reference values. Gini Index is generally used with decision trees to calculate the impurity degrees of splitting (Hastie et al. 2001). Briefly, the method Gini Index would be used to find out a split point for any data to discriminate the outcomes below and above this split points in terms of homogeneity. Herewith the outcomes were the adverse events and the data were the AUC and Cmax.

Gini Index given in Eq. (1) should be calculated for every candidate *v* values which are generally original Cmax values. In our study, arithmetic mean was calculated for Cmax values of test and reference drugs for each BE study and each adverse event exists/not exists (1/0) class information was added correspondingly, then values were sorted in increasing order and candidate split positions were identified by taking the mid-points between two adjacent sorted Cmax values. For each candidate *v* value, the dataset was scanned once to count the number of records with Cmax lower than or higher than $v$. The Gini Index for each candidate *v* was then computed and the one that gives the lowest Gini value, which indicated the best splitting position, was chosen (Hastie et al. 2001; Tan et al. 2006).

1 $$GINI(t) = 1 - \sum\limits_{j} {[p(j|t)]^{2} }$$

In this study, j is the index of class and it can be either adverse event exists or not exists, t is the value at the current split position, therefore *p*(*j*|*t*) denotes the fraction of records belonging to class j at a given split position t.

#### An example for the Gini Index method application to a single BE study

In order to explain the Gini Index calculation more clearly, detailed calculation steps of one of the BE studies can be given as an example. As stated earlier, there were 261 BE studies and only 63 of them had Cmax and also AUC values available. These studies were indexed from 1 to 261. BE study number 121 which included 48 volunteers was chosen as an example. BE study number 121 was an open-label, randomised, single oral dose, two-period, cross-over trial and conducted in order to assess the BE of two different tablet formulations of repaglinide 2 mg which is used as an antidiabetic. For this study, the arithmetic mean of the test and reference Cmax values were calculated for each volunteer and adverse event exists/not exists (1/0) class information was added correspondingly. After constructing a submatrix with 48 rows and two columns (one for the mean of the test and reference values, one for the adverse event class information 1 or 0) for the study 121, the subset was sorted in terms of the mean column in increasing order. To find candidate split positions, mid-points of two adjacent sorted mean Cmax values were calculated. Once the candidate split values were obtained, the Gini Index for each of them was computed using Eq. (1) given before. To get the highest information gain, the lowest Gini value should be chosen as the best splitting position. According to this rule, the lowest Gini is found at position 68.8225 for study 121. The number of adverse events which were corresponded to Cmax values lower than 68.8225 is 6 and higher than 68.8225 is 19. This process has been done for each BE study, therefore at the end of the calculations we had different split points.

## Results

### Overall demographic information of subjects and design of the BE studies

The dataset consisted of 261 BE studies with 7828 volunteers who attended these studies between the years 2000 and 2013. After preprocessing and cleaning the data, 5498 volunteers with their demographic information such as age, height, weight and body mass index (BMI) were obtained. All these demographic information is summarized in the Table 1. Out of 261 BE studies 240 studies were carried out as classical two periods, one study was partial replicated three periods, one study was four periods crossover replicated one, and 19 of them were designed as four periods fasting-fed study.

**Table 1:** The demographic information of 5498 volunteers

Variable	Mean ± SD	Min–max
Age	26.55 ± 2.96	21.12–49.08
Height	174.33 ± 1.53	167.83–179.08
Weight	71.22 ± 2.12	66.87–77.71
BMI	23.44 ± 0.68	21.81–25.62

*BMI* body mass index

### APIs and drug subgroups in the pooled BE studies

In 261 BE studies, 109 different APIs were determined (Additional file 1). It was also observed that all drugs, classified as level 1 according to Anatomical Therapeutic Classification (ATC), can be divided into eight different subgroups based on the system they affected. The distribution of 261 studies according to the drug subgroups revealed that 66 studies were in the group of cardiovascular system drugs while each genito-urinary system and sex hormones subgroup, and antineoplastic and immunomodulating agent subgroup were represented with two studies (Table 2).

**Table 2:** Frequency of adverse events according to the drug subgroups

Drug subgroup (ATC classes)	Number of studies	Number of volunteers	Number of adverse events	Adverse event frequency per volunteer (%)
Cardiovascular system	66	2095	355	16.9
Antiinfectives for systemic use	56	1409	126	8.9
Musculo-skeletal system	46	1595	108	6.8
Alimentary tract and metabolism	42	1280	276	21.6
Nervous system	39	1121	681	60.8
Respiratory system	8	228	27	11.8
Genito-urinary system and sex hormones	2	48	48	100
Antineoplastic and immunomodulating agents	2	52	21	40.4
Total	261	7828	1642	21

*ATC* anatomical therapeutical classification (Investigation 1994)

### Adverse event classification according to the frequency, drug subgroups and type

Table 2 shows that there were 1642 adverse events (1638 adverse events and four serious adverse events) observed in the 261 studies. The frequency of the adverse events was calculated as 6.29 per study and 0.21 per volunteer (Table 2). Distribution of the adverse events was also examined in terms of the eight drug subgroups. In genito-urinary system and sex hormones subgroup 48 adverse events with the highest frequency (100 %) were found and in musculo-skeletal system drug subgroup 108 adverse events with the lowest frequency (6.8 %) were found. Thirty nine studies with 1121 volunteers were conducted for the nervous system drugs. 681 adverse events were observed. Frequency of adverse events with nervous system drugs was found as 60.8 % per volunteer (Table 2).

Following the classification of the adverse events, 104 different adverse event types were observed. According to the Table 3, the most frequently observed adverse events were headache with 32.1 %, somnolence with 15.8 %, nausea with 11.9 %, dizziness with 7.1 % and vomiting with 3.3 %, respectively (Table 3). Other adverse event types (99) had the frequency of 29.8 % (Additional file 2). Two hundred and forty out of 261 BE studies were conducted as two period design, while one of them designed as three period and 20 of them designed as four period. Thousand six hundred and twenty eight adverse events out of 1642 were seen in the period I and period II (Table 3).

**Table 3:** The distribution of the most frequent adverse events related to the BE study designs

	BE study periods				Before dosing/washout^a^	Total
	I	II	III	IV		
Adverse events (total)	*870*	*758*	*6*	*1*	*7*	*1642*
Adverse events types						
Headache	262	260	5	–	–	527
Somnolence	130	129	–	–	–	259
Nausea	100	93	1	1	–	195
Dizziness	61	55	–	–	–	116
Vomiting	34	19	–	–	1	54
Other adverse events	283	202	–	–	6	491

^a^Elimination process of drug from the volunteer to ensure that drug concentrations are below the lower limit of bioanalytical quantification in all volunteers at the beginning of the second period (Cathcart et al. 2010)

In the pooled data there were four serious adverse events namely right foot fracture during entecavir BE study, laceration of achilles tendon while playing football match during tenofovir study, fall and traumatic scalp injury during quetiapine study, death in traffic accident during washout period of zafirlukast study.

### Distribution of adverse events according to Cmax and AUC split points by Gini Index

The Gini Index evaluations revealed split point for each 63 BE studies in Cmax and also AUC values to display possible relationships between them and adverse event exist/not exist knowledge. As a result of the Gini Index approach, each BE study had its own split point for both Cmax and AUC, respectively. It can be generalized that split point that obtained by Gini calculation can be considered as a cut off for the frequency of the adverse events. Thus, the number of adverse events obtained below the split point is lower than the number of adverse events obtained above the split point. To avoid biased generalization, the number of subjects (volunteers) was also considered and summarized in the Table 4. The overall evaluation of the split point and related probabilities which are calculated by the number of adverse events/the number of volunteers ratio for both site (below and above) of the split point can be seen from Table 4. According to results given in this table, both numbers of adverse events and probabilities, which were obtained by considering number of subjects, supported the generalization mentioned above and given in italic font for both Cmax and AUC.

**Table 4:** Overall results observed above/below split points for Cmax and AUC values

	Numbers of adverse events		Numbers of subjects		Probabilities	
	Below the split point	Above the split point	Below the split point	Above the split point	Below the split point	Above the split point
Cmax	141	210	854	1138	0.165	*0.185*
AUC	116	235	861	1131	0.135	*0.208*

Probabilities are calculated by number of adverse events below (above) the split point/number of subjects below (above) the split point, and higher probabilities given in italic font

Similar analyses were also done in terms of the ATC subgroups both for Cmax and AUC and the detailed results are shown in Tables 5 and 6. According to split point approach, the occurrence of the adverse events below the split point obtained by Gini Index was less probable than the occurrence of the adverse events above the split points. In order to make unbiased evaluation, probabilities were calculated for each ATC groups with respect to the number of adverse events and number of volunteers for both site of the split point obtained by Gini Index. It can be easily seen from Tables 5 and 6, most of the results obtained for ATC groups are supported our claim. In these tables, higher probability among the probabilities obtained for both sites of split point is given in italic font.

**Table 5:** Results observed above/below split points for Cmax values in terms of ATC subgroups

Cmax	Number of adverse events		Number of subjects		Probabilities	
ATC subgroups	Below split point	Above split point	Below split point	Above split point	Below split point	Above split point
Cardiovascular system	17	17	173	154	0.098	*0.110*
Antiinfectives for systemic use	11	14	162	249	*0.068*	0.055
Musculo-skeletal system	11	10	136	188	*0.081*	0.053
Alimentary tract and metabolism	27	66	109	241	0.248	*0.274*
Nervous system	70	67	249	188	0.281	*0.356*
Respiratory system	2	9	13	83	*0.154*	0.108
Genito-urinary system and sex hormones	3	27	12	35	0.25	*0.771*

Probabilities are calculated by number of adverse events below (above) the split point/number of subjects below (above) the split point, and higher probabilities given in italic font

**Table 6:** Results observed above/below split points for AUC values in terms of ATC subgroups

AUC	Number of adverse events		Number of subjects		Probabilities	
ATC subgroups	Below split point	Above split point	Below split point	Above split point	Below split point	Above split point
Cardiovascular system	15	19	216	111	0.069	*0.171*
Antiinfectives for systemic use	10	15	164	247	*0.061*	*0.061*
Musculo-skeletal system	7	14	81	243	*0.086*	0.058
Alimentary tract and metabolism	19	74	126	224	0.151	*0.330*
Nervous system	50	87	218	219	0.229	*0.397*
Respiratory system	10	1	41	55	*0.244*	0.018
Genito-urinary system and sex hormones	5	25	15	32	0.333	*0.781*

Probabilities are calculated by number of adverse events below (above) the split point/number of subjects below (above) the split point, and higher probabilities given in italic font

Besides our generalization about split point is supported by overall results given in Table 4 for both Cmax and AUC, probabilities of the drugs from cardiovascular system, alimentary tract and metabolism, nervous system and genito-urinary system and sex hormones are also obtained above the split point as seen in Tables 5 and 6 and these results also support our generalization. Thus, corresponding split points can be used as the reference values for the future BE studies.

Among 261 BE studies only 63 studies with 1992 volunteers were available with the adverse event data of both test and reference drugs. Accordingly, 352 adverse events were observed after administration of reference drugs and 352 adverse events were seen after administration of test drugs.

## Discussion

Current study could be briefly described as the pooled analysis of BE studies with regards to the adverse events. Although the BE studies are widely being conducted all over the world, a research on overall adverse event specific evaluations were not found. Research on the adverse reactions as a post-marketing safety survelliance often takes place in the scientific literature (Lazarou et al. 1998; Sibille et al. 1998; Baker et al. 2004) while BE studies were mostly focused on the pharmacokinetics, assessing the relationship between genetic polymorphisms, and safety issues of the single test (generic) and reference (originator) drugs (Zhang et al. 2006; Zhang et al. 2007; Cho et al. 2009; Esseku et al. 2013; Gao et al. 2015; Mak et al. 2015). Therefore, our study has the originality by revisiting the BE studies of 13 years period belonging to one of the CRO’s database (N-CRO) in Turkey, regarding the frequency, type, drug subgroups, cut off (split points using Gini Index) values and probable causes of adverse events.

The pooled BE dataset also evaluated the age, BMI, and sex of the 5498 volunteers. The range of age showed that younger people (min. 21.12–max. 49.08) were more eager to be involved in the studies, which could be explained by the socioeconomical status. While it was possible for both of the sexes to be involved in the BE studies of N-CRO, all the volunteers were male. This could be related to the sociocultural properties of a developing country. Reluctance of females to attend bioequivalence studies in Turkey makes restrictor effect to number and representativeness of BE studies, especially for hormone containing drugs (e.g., oral contraceptives). Moreover, pharmacokinetic parameters like Cmax and AUC for some drugs, thereby adverse events are well-known to show differences between male and female individuals (Gandhi et al. 2004; Soldin and Mattison 2009). Even tough to involve only male volunteers to the BE studies is not against to the regulations framed by the national and international guidelines (Investigation 1994; EMA 2010), for a CRO it could be a limitation for evaluating pharmacodynamics and pharmacokinetic differences due to gender in BE studies. We could suggest raising public awareness with the use of media in order to involve both sexes to the BE studies.

In the pooled BE study database, the highest frequency of adverse effects was reported in genitourinary system and sex hormones involving tadalafil and sildenafile containing drugs among the eight drug subgroups. Likewise, drugs which contain tadalafil and sildenafile also attracted attention in the post-marketing adverse event reports. (Lowe and Costabile 2012). Genitourinary system and sex hormones’ were followed by nervous system, and antineoplastic drugs indicating relatively high adverse event frequencies when compared to the other subgroups (Leung et al. 2012; Pages et al. 2014).

The finding of no difference between test and reference drugs in terms of adverse events frequency is a valuable result for safety of generic drugs while investigating a population of 1992 volunteers.

The first five highest frequent adverse events (headache, somnolence, nausea, dizziness, vomiting) in the BE studies were related with nervous system and gastrointestinal system. Probably, those adverse events could be based on the drug itself. In the adverse event forms most of the adverse events were recorded as irrelevant to the drugs used in the BE studies. While it can be speculative, some of the adverse events could be assumed to be triggered with psychological stress (Mayer 2000; Mönnikes et al. 2001; Nash and Thebarge 2006; Leistad et al. 2006; Cathcart et al. 2010). Some studies suggested that headache and functional gastrointestinal disorders might have psychological stress origin (Mayer 2000; Mönnikes et al. 2001; Leistad et al. 2006; Nash and Thebarge 2006; Cathcart et al. 2010) while fatigue and somnolence might have psychosomatic origin (Shorter 1993; Loge et al. 1998; Taylor et al. 2003). It can be speculated that to be involved in a BE study, giving biological specimen such as blood all day long, and having no other activity could be unusual to the daily routine of a person and it is possible to trigger some adverse events based upon psychological stress (Shorter 1993; Loge et al. 1998; Mayer 2000; Mönnikes et al. 2001; Taylor et al. 2003; Nash and Thebarge 2006; Leistad et al. 2006; Cathcart et al. 2010). Thus, the physical and social environment should be examined, and if necessary, in order to reduce the psychological stress that leads to adverse events, the prevailing conditions in the centers could be improved.

The investigators recorded serious adverse events (right foot fracture, laceration of achilles tendon, fall and traumatic scalp injury, death in traffic accident) to serious adverse event forms, applied specified treatment protocol to the volunteer where available, and informed both Ethical Committee and Turkish Ministry of Health. The investigators noted that none of the serious adverse events observed were showed relationship with the administrated drug in the BE studies. Our study could provide more robust and effective data about adverse events if investigators of Clinical Trial Centers have used MedDRA dictionary while classifying adverse events.

Inter-individual differences as genetic polymorphism are known to affect the drug metabolism and pharmacokinetic parameters. For example, Cmax and AUC parameters could be significantly different between poor drug metabolisers and extensive drug metabolisers resulting in toxic responses or lack of therapeutic effectiveness (Karaźniewicz-Łada et al. 2014; Al-Gahtany et al. 2014; Fowler et al. 2015). Therefore, it is possible to claim the crucial role of the inter-individual differences in the adverse events reported in the BE studies.

As we dealed with a pooled dataset of single BE studies covering 13 years, it was an opportunity to use Cmax and AUC values in order to obtain split points (cut off levels) to estimate adverse event frequencies below and above them. Gini Index is adopted from finance, where it is used to measure the inequality of wealth (Cichosz 2015). It is commonly used with well-known classification method named decision trees which h as wide usage area including finance, medicine, social sciences etc. (Tan et al. 2006). Thus, our group utilized Gini Index approach for the first time in BE studies to show the likely individual pharmacokinetic differences in the adverse event occurence with promising results. Eventually, the frequency of adverse events of the volunteers whose Cmax and AUC values above the obtained split points was found to be higher than that of the ones below. Since there were no additional data available indicating volunteers’ inter-individual differences in the dataset, split point estimation according to the Cmax and AUC values could be offered as a promising tool. Also, this approach could provide valuable outcomes when combined with genetic polymorphism data. As the pharmacokinetic parameters were strongly influenced by interindividual differences, we suggest using Gini Index in overall pharmacokinetic and pharmacovigilance evaluations in order to assess the pharmacogenomic differences via adverse event outcome.

The Gini Index based split points, with regards to the AUC and Cmax values for adverse event distribution, could be suggested for the future BE studies carried out for similar drugs to predict the frequency of likely adverse events.

However it should be emphasized that it has been found as a limitation to have only 63 BE studies (1992 volunteers) out of 261 studies with reachable Cmax and AUC data in our overall evaluation. The reason was N-CRO started to use new recording system for pharmacokinetic parameters at 2006. Previously, Cmax/AUC and some other parameters were not transferrable from N-CRO’s database.

## Conclusions

The retrospective review of 13 years period of pooled BE studies of N-CRO revealed that the design of the studies and the demographic properties of the volunteers were consistent with both national and international guidelines. However, in the BE studies only male volunteers were involved. It can be suggested to promote the involvement of females by raising the public awareness through media. The statistical approach named Gini Index used for the first time in order to determine the role of inter-individual differences in the adverse event occurrence and it is found to be a valuable tool to recommend for similar future studies. It could be more revealing to have the Cmax and AUC of all of the 261 BE studies. As a conclusion, our study proposed that centralized monitoring of pooled BE studies with regards to the adverse events could support drug safety studies and provide a basis for post-marketing pharmacovigilance studies.

## Additional files

10.1186/s40064-016-2527-4 APIs of BE studies.
10.1186/s40064-016-2527-4 Adverse event types in BE studies.
