# Supplementary material for: Pooled bioequivalence study database from Turkey: characterization of adverse events and determination of split points based on Gini Index as a promising method
Source: Springerplus. 2016 Jun 13;5(1):709. doi: 10.1186/s40064-016-2527-4 (PMC4906091; doi:10.1186/s40064-016-2527-4)
Supplement: Supplementary file 1 — 10.1186/s40064-016-2527-4 APIs of BE studies. [file 40064_2016_2527_MOESM1_ESM.docx]

| Group Number | Drug Subgroups (ATC Level 1) | API (n) |
| --- | --- | --- |
| 1 | [Antiinfectives](https://en.wikipedia.org/wiki/Antiinfective) for systemic use | Adefovir, Amoxicillin, Amoxicillin + Clavulanic acid *  Azithromycin, Entecavir, Fluconazole, Fusidic acid, Itraconazole, Clarithromycin, Lamivudine, Levofloxacin, Moxifloxacin, Nitrofurantoin, Ornidazole, Oseltamivir, Ribavirin, Cefaclor, Cefalexin, Cefixime, Cefpodoxime proxetil, Cefuroxime axetil, Ciprofloxacin, Spiramycin, Tenofovir, Terbinafine, Tetracycline (26) |
| 2 | [Genito-urinary system](https://en.wikipedia.org/wiki/Genito-urinary_system) and [sex hormones](https://en.wikipedia.org/wiki/Sex_hormone) | Sildenafil, Tadalafil (2) |
| 3 | [Cardiovascular system](https://en.wikipedia.org/wiki/Cardiovascular_system) | Amlodipine, Atorvastatin, Enalapril, Enalapril+HCT*, Ezetimibe, Indapamide, Irbesartan, Irbesartan+HCT*, Calcium Dobesilate, Candesartan, Candesartan+HCT*, Captopril, Carvedilol, Kinapril+HCT*, Losartan, Losartan+HCT*, Olmesartan, Olmesartan+HCT*, Perindopril, Perindopril+Indapamid*, Ramipril, Ramipril+HCT*, Rosuvastatin, Silazapril, Silazapril+HCT*, Trimetazidine, Valsartan, Valsartan+HCT* (28) |
| 4 | [Musculo-skeletal system](https://en.wikipedia.org/wiki/Musculo-skeletal_system) | Alendronate, Dexketoprofen, Diflunisal, Diclofenac sodium, Etodolac, Flurbiprofen, Ibuprofen+pseudoephedrine HCI*, Meloxicam, Naproxen sodium, Refecoxib, Selecoxib, Tenoxicam, Thiocolchicoside, Thiocolchicoside +Flurbiprofen* (14) |
| 5 | [Alimentary tract](https://en.wikipedia.org/wiki/Alimentary_tract) and [metabolism](https://en.wikipedia.org/wiki/Metabolism) | Famotidin, Gliclazide, Glimepiride, Granisetron, Lansoprazol, Metformine,Metformine+Glibenclamide*, Metformine+Vildagliptin*, Nateglinide, Pantoprazol, Pioglitazone, Ranitidine, Repaglinide, Metformine+Rosiglitazone*, Sibutramine (15) |
| 6 | [Nervous system](https://en.wikipedia.org/wiki/Nervous_system) | Aripiprazole, Donepezil, Duloxetine, Escitalopram, Fluoxetin, Quetiapine, Levetiracetam, Mirtazapine, Olanzapine, Opipramol, Paroxetin, Rasagiline, Risperidone, Sertralin, Citalopram, Topiramat, Zolmitriptan (17) |
| 7 | [Respiratory system](https://en.wikipedia.org/wiki/Respiratory_system) | Desloratadine, Fexofenadine, Loratadine, Montelukast, Zafirlukast (5) |
| 8 | [Antineoplastic](https://en.wikipedia.org/wiki/Antineoplastic) and [immunomodulating](https://en.wikipedia.org/wiki/Immunomodulator) agents | Erlotinib, Imatinib (2) |

*:Fixed dose combination
