# Supplementary material for: Pooled bioequivalence study database from Turkey: characterization of adverse events and determination of split points based on Gini Index as a promising method
Source: Springerplus. 2016 Jun 13;5(1):709. doi: 10.1186/s40064-016-2527-4 (PMC4906091; doi:10.1186/s40064-016-2527-4)
Supplement: Supplementary file 2 — 10.1186/s40064-016-2527-4 Adverse event types in BE studies. [file 40064_2016_2527_MOESM2_ESM.docx]

| 1. Gastric pain |
| --- |
| 1. Lethargy |
| 1. Vertigo |
| 1. Convulsion |
| 1. Itching |
| 1. Syncope |
| 1. Diarrhea |
| 1. Jaw pain |
| 1. Dry Throat |
| 1. Hypotension |
| 1. Arthralgia |
| 1. Bilateral knee swelling |
| 1. Fever |
| 1. Plantar burning sense |
| 1. Common cold |
| 1. Dispnea |
| 1. Cough |
| 1. Bradicardia |
| 1. Sore throat |
| 1. Numbness on jaw |
| 1. Dispepsia |
| 1. Abdominal pain |
| 1. Dry mouth |
| 1. Angina pectoris |
| 1. Sedation |
| 1. Palpitation |
| 1. Irritation |
| 1. Tachycardia |
| 1. Enteritis |
| 1. Infectous disease |
| 1. Pruritus |
| 1. Squamation |
| 1. Tonsillitis |
| 1. Fatique |
| 1. Epileptic convulsion |
| 1. Hypotensive attack |
| 1. Chest pain |
| 1. Numbness on hand and foot |
| 1. Euphoria |
| 1. Jaw spasm |
| 1. Weakness 2. difficulty of breathing and felt breathless 3. loose stools 4. skin rush on legs 5. Renal colic 6. allergic eruption on back and antecubital area 7. blurred vision 8. hypoglycemic symptoms 9. tremor on hands 10. trembling 11. Metallic/bad taste in mounth 12. Sweating 13. Hypogylcemia 14. Sweating at night 15. Low back pain 16. Pyrosis 17. İtching on hands 18. Stomach ache 19. Epigastric pain 20. İnflommation on foot 21. Nocturia 22. Anxiety 23. Toothache 24. Lumbargia 25. Abdominal distension 26. Constipation 27. Drowsiness 28. Myalgia at back 29. Epigastric burn 30. Nose bleeding 31. Anorexia 32. Pain in knees 33. Pain in limbs 34. Softening in feces 35. Night mare 36. Flu like symptoms 37. Pain in shoulder 38. Heart burn 39. İncreased salivation 40. Myalgia 41. Stinging pain İn lover left Chest 42. Lost blood due to accidentally opening the tap of the catheter 43. Increased serum ALT level 44. Soft defaction 45. Pain on lumbal region 46. SGPT and GGT elevation 47. İnsomnia 48. Tingling in toes on left 49. sleepiness 50. feel cold 51. flatulance 52. Increased Sexual desire 53. Taste Disorder 54. Right hand index finger incision due to door pack 55. Right leg pain due to trauma 56. S.A.E Right foot fracture 57. S.A.E. Left Achiles Tendon Rupture(during playing football match) 58. S.A.E Had fallen due to hypotension and vertigo, and occurred a traumatic scalp injury 59. S.A.E.died in a traffic accident during washout period |
